# Sugary beverages intake and risk of chronic kidney disease: the mediating role of metabolic syndrome

**DOI:** 10.3389/fnut.2024.1401081

**Published:** 2024-11-26

**Authors:** Xiao-Yu Dai, Xiang-Yu Chen, Li-Na Jia, Xiao-Tong Jing, Xiao-Yan Pan, Xing-Yu Zhang, Zhong Jing, Jin-Qiu Yuan, Qiang-Sheng He, Li-Ling Yang

**Affiliations:** ^1^Department of Nephrology, Mianyang Central Hospital, School of Medicine, University of Electronic Science and Technology of China, Mianyang, China; ^2^Jiange People's Hospital, Jiange, Sichuan, China; ^3^Department of Nephrology, Meishan Second People's Hospital, Meishan, Sichuan, China; ^4^Central Sterile Supply Department, Mianyang Central Hospital, School of Medicine, University of Electronic Science and Technology of China, Mianyang, China; ^5^School of Nursing, Wuhan University, Wuhan, China; ^6^Department of Nephrology, Mianyang 404 Hospital, Mianyang, China; ^7^Clinical Research Center, The Seventh Affiliated Hospital, Sun Yat-sen University, Shenzhen, Guangdong, China; ^8^Scientific Research Center, The Seventh Affiliated Hospital, Sun Yat-sen University, Shenzhen, Guangdong, China

**Keywords:** chronic kidney disease, sugar-sweetened beverages, artificially sweetened beverages, natural juices, mediation analyses

## Abstract

**Background:**

Although several studies linked the sugary beverages to chronic kidney disease (CKD), the role of different types of sugary beverages in the development of CKD remained inconsistent. This study aimed to examine the associations of sugar-sweetened beverages (SSBs), artificially-sweetened beverages (ASBs), and natural juices (NJs) with CKD risk, and assess the extent to which the associations were mediated through metabolic syndrome (MetS).

**Methods:**

This is a prospective analysis of 191,956 participants from the UK Biobank. Participants with information on beverage consumption and no history of CKD at recruitment were included. Daily consumptions of SSBs, ASBs and NJs were measured via 24-h dietary recall. Cox models were fitted to calculate the hazard ratios (HRs) and confidence intervals (CIs) of sugary beverages intakes on CKD risk. The causal mediation analyses were conducted to investigate whether MetS explained the observed associations.

**Results:**

We documented 4,983 CKD cases over a median of 10.63 years follow-up. Higher consumption of SSBs and ASBs (>1 units/d compared with none) was associated with an elevated risk of CKD (HR: 1.45; 95% CI: 1.30–1.61, *P*-trend < 0.001 for SSBs and 1.52, 95% CI: 1.36–1.70 for ASBs). In contrast, we observed a J-shaped association between NJs and CKD with the with lowest risk at 0–1 unit/day (0–1 unit/d vs. 0, HR 0.86; 95% CI 0.81–0.91). The proportions of the observed association of higher intakes of SSBs and ASB with CKD mediated by MetS were 12.5 and 18.0%, respectively.

**Conclusions:**

Higher intakes of ASBs and SSBs were positively associated with the development of CKD, while moderate consumption of NJs was inversely associated with CKD risk. More intensified policy efforts are warranted to reduce intake of SSBs and ASBs for CKD prevention.

## Introduction

The deleterious effects of added sugars have been established for multiple health outcomes, and recognized as major risk factors by several health authorities, such as World Health Organization (WHO) (1). As the main source of added sugars in the diet, sugar- sweetened beverages (SSBs) have been linked to with several chronic diseases, such as obesity, type 2 diabetes, certain cancers, cardiovascular disease, and mortality (2–5). A recent study based on the Global Burden of Disease (GBD) 2019 showed that the number of deaths for chronic non-communicable diseases attributed to high SSBs increased from 149,988 in 1990 to 242,218 in 2019, representing a 61% increase (6). Artificially sweetened beverages (ASBs) and natural juices (NJs, 100% pure fruit or vegetable juices) are considered alternatives to SSBs, but their long-term effects on human health are still controversial (7–12). Evidence from systematic reviews and meta-analyses has suggested that higher consumption of ASBs was associated with risk of obesity, hypertension, type 2 diabetes, and all-cause mortality (10–12).

Chronic Kidney Disease (CKD) is a major global public health concern with an increasing prevalence and massive socio-economic impact (13). The study based on GBD data showed that the global prevalence of CKD was 9.1% in 2017, causing 35.8 million disability-adjusted life years and 1.2 million deaths (13). CKD is a complex condition that results from various genetic, environmental, and behavioral factors, such as unhealthy lifestyles, hypertension, obesity, and diabetes mellitus (14). Although higher consumption SSBs and ASB have been linked to CKD risk factors (i.e., obesity, hypertension, and diabetes), their prospective associations with CKD have not yet been well-established due to inconsistent findings of previous studies (15–17).

Previous studies examining the associations of the consumption SSBs and ASBs with CKD risk have yielded contradictory and inconsistent findings (15–19). For example, two prospective studies in the US and Iran found positive associations between higher consumption SSBs and the risk of incident CKD (19, 20), while other studies based on the Atherosclerosis Risk in Communities and Nurses' Health Study in U.S. showed no significant association of SSBs with CKD risk (21, 22). A meta-analysis in 2021 indicated that higher consumption SSBs or ASBs was associated with a non-significant increased risk of CKD (SSBs, RR 1.30, 95% CI 0.88–1.94, *n* = 6 studies; ASBs, RR 1.40, 95% 0.65–3.02, *n* = 3 studies) (15). However, the studies found that the risk of CKD significantly increased if the SSBs or ASBs intake exceeded seven servings per week (15). Differences in study design and participants, sample sizes, definitions and measurements of exposure might explain the inconsistent findings, making it difficult to draw definitive conclusions regarding beverages consumption and CKD risk.

Furthermore, the biological pathway underlying the association between beverage consumption and CKD risk remains unclear. Given the causal role of metabolic risk factors such as obesity and hypertension in the CKD development, and the well-established association of SSBs or ASBs intake with these metabolic risk factors, the substantial mediation of the SSBs or ASBs effect through the metabolic risk factors seems biologically plausible. However, no study to date has examined the potential mediation effect of metabolic risk.

In the present study, we conducted a prospective analysis of the UK Biobank cohort to evaluate the long-term association of SSBs, ASBs, and NJs with incident CKD. We further estimated to what extent the metabolic syndrome (MetS) and its individual components mediated the observed associations of different types beverages with CKD risk.

## Methods

### Study population

The UK Biobank is an ongoing large-scale prospective cohort study that contains in-depth genetic and health information from over 500,000 UK adults aged 40–69 years. Between 2006 and 2010, eligible participants were invited to attend the nearest assessment centers and were asked to complete a touchscreen questionnaire, a brief interview and a range of physical measurements, as well as provide biological samples. Details of the study design, survey methods, data collection and follow-up for the UK Biobank could be found elsewhere (23). The study received full ethical approval from the North West Multi-center Research Ethics Committee, and all participant provided a written informed consent prior to data collection.

For the present study, we included 210,961 participants who completed at least one online 24-h diet recall questionnaire. We excluded participants CKD (*n* = 4,271), or with cancers diagnosis (*n* = 14,214) prior to baseline, and those who subsequently withdrew from the study (*n* = 520). The final analysis dataset comprised 191,956 participants for the primary analysis (Supplementary Figure 1), with 53,701 participants having baseline data on MetS and sugary beverages used in the secondary mediation analyses.

### Exposure assessment

Dietary information was assessed using the Oxford WebQ, a web-based 24-h dietary assessment tool, which participants completed via an online questionnaire on five occasions between 2009 and 2012 (Supplementary Figure 1). In this assessment, participants were asked: “*how much of low calorie or diet drinks, carbonated drinks, fruit drinks (including J2O, squash, or cordial), natural orange juice, natural grapefruit juice, and other natural fruit/vegetable juice, did you drink yesterday?*” Response options included 0, 0.5, 1, 2, 3, 4, 5, and more than 6 units. In the current study, SSBs included fizzy drink and squash, ASBs were defined as low-calorie drinks, and NJs included orange juice, grapefruit juice, and other pure fruit or vegetable juices (24, 25). We calculated the mean beverage intake of participants who completed more than one questionnaire. Participants were categorized into three groups based on consumption of each beverage as follows: 0, 0–1, >1 unit per day, respectively.

### Assessment of CKD

The main outcome of the study was incident CKD, identified linkage to electronic health records based on the International Classification of Diseases (ICD)-10 codes (N18). We utilized CKD variables provided by the UK Biobank, which integrated information from various data sources, including primary care, hospital admissions, self-report, and death registers. Details of the methods used to identify CKD can be found on the UK Biobank website (26).

### Mediators

The main mediator variable of this study was MetS, defined as the presence of three or more of the following components: central obesity, hypertriglyceridemia, reduced high-density lipoprotein cholesterol, elevated blood pressure and hyperglycemia (27, 28). These components were assessed at baseline and defined according to the NCEP ATP III definition (see Supplementary Table 1). Given the well-established association between Hyperuricemia and CKD (29), a second MetS mediator was derived, which also included Hyperuricemia as part of MetS. To ensure comparability, the same definition of MetS was used (i.e., 3 or more metrics met).

### Covariates

Covariate information was collected at baseline using a self-completed touchscreen questionnaire. Sociodemographic factors (age, sex, ethnicity, income, and education level), lifestyle behaviors (including smoking status, alcohol consumption frequency, physical activity, fruit and vegetable intake, red and process meat intake, sleep time and multivitamin intake) and medication use (i.e., aspirin, non-aspirin non-steroidal anti-inflammatory drugs [NSAIDs], and proton-pump Inhibitors) were self-reported. Index of multiple deprivation, a composite score of area-based socioeconomic status, was directly obtain from UK biobank. Additional details regarding the derivation of these variables are provided in Supplementary Table 1.

### Statistical analysis

Follow-up time in person-years was from the date of the first available 24-h questionnaire to the to the date of first diagnosis of CKD, death, or end of follow-up (31 October 2021), whichever came first. Cox proportional hazard models were fitted to estimated hazard ratios (HRs) and 95% confidence intervals (CIs) for the effects of SSBs, ASBs, and NJs consumption on the risk of CKD. In the basic model, we stratified the analyses jointly by sex and age (37–50, 50–60, and ≥60 years). We further adjusted for ethnicity, income, education level, smoking status, alcohol consumption, physical activity, fruit and vegetable intake, red and processed meat intake, sleep time, and multivitamin intake in the multivariable-adjusted model 1. Additionally, we adjusted for total sugar intake, total energy, and two other beverages in the multivariable-adjusted model 2. Proportional hazards assumption was checked using Schoenfeld's tests and no violation was shown.

We used restricted cubic splines to explore potential non-linear associations between sugary beverage intake and CKD risk. Substitution analysis was conducted to investigate potential associations between substituting each unit of the three types of sugary beverages with each other and incident CKD. Additionally, we evaluated whether the associations between sugary beverages and CKD risk were modified by sex, age, household income, smoking, drinking status, hypertension, hyperglycemia, MetS status, and the predicted 5-year risk of CKD by testing the interactions of each beverage and covariate and conducting subgroup analyses (30). Several sensitivity analyses were performed to check the robustness of the primary results. First, we lagged the exposure for 2 years to minimize reverse causality. Second, participants with cardiovascular disease at baseline were excluded to minimize the potential influence of the medical condition. Third, the models were further adjusted for energy from beverages to assess if the associations were independent of energy intake. Fourthly, the models were further adjusted for estimated glomerular filtration rate (eGFR) to account for the influence of kidney function at baseline. Lastly, we used the Fine-Gray subdistribution hazard model to account for the competing risk of death.

Mediation analyses were performed to evaluate the role of MetS and its individual components as a mediator of the relationship between beverage consumptions and CKD risk. We used the counterfactual framework approach developed by Valeri and VanderWeele (31), which decomposes the total effect into natural direct and indirect effects and accommodates the interaction of the primary exposure with the mediator of interest. This method has been extended to the context of time-to-event analysis with Cox regression (32). As VanderWeele described (31, 32), the exposure-mediator and exposure-outcome relations were modeled with linear and Cox regression, respectively, adjusted for covariates as in the model above. We estimated the direct association of SSBs, ASBs, and NJs on CKD risk independent of MetS, the indirect association of beverage consumptions mediated through MetS, and the proportion of the total association mediated by MetS. All statistical tests were two-tailed. Statistical analyses were performed using the SAS (release 9.4; SAS Institute Inc) and R software (version 3.5.0).

## Results

Among 191,956 participants included in the analysis, 62,453 (32.6%) drank SSBs, 22,790 (11.9%) drank ASBs, and 63,318 (33.0%) drank NJs. Participants who consumed more SSBs were more likely to be younger, male, deprived, smokers, had a higher daily total energy and sugar intake, and a higher prevalence of MetS (Table 1). Meanwhile, those who consumed more ASBs tended to be younger, female, deprived, more highly educated, with a higher prevalence of medication use (e.g., aspirin, proton pump inhibitors) and MetS (Supplementary Table 2). Additionally, NJs consumers tended to be male, more educated, have higher income, be non-smokers, and have higher daily total energy and sugar intakes (Supplementary Table 3).

**Table 1:** Baseline characteristics of participants by consumption of sugar-sweetened beverages.

	**Sugar-sweetened beverages intake, unit/day**		
	**0**	**0–1**	>**1**
Number of participants	129,413	49,594	12,949
Mean (SD) age, years	56.63 (7.83)	55.91 (8.02)	53.60 (8.14)
Male	56.802 (43.9)	23,464 (47.3)	7,224 (55.8)
*N* (%) white	124,336 (96.1)	47,176 (95.1)	12,051 (93.1)
Mean (SD) index of multiple deprivation	15.20 (12.12)	15.38 (12.38)	17.40 (13.86)
**Education**			
Less than high school	11,676 (9.0)	3,985 (8.0)	1,136 (8.8)
High school or equivalent	48,385 (37.4)	19,189 (38.7)	5,567 (43.0)
College or above	69,352 (53.6)	26,420 (53.3)	6,246 (48.2)
**Household incomey**			
Low	17,459 (13.5)	6,530 (13.2)	2,001 (15.5)
Medium	60,685 (46.9)	23,856 (48.1)	6,215 (48.0)
High	37,838 (29.2)	14,269 (28.8)	3,461 (26.7)
Unknown/missing	13,431 (10.4)	4,939 (10.0)	1,272 (9.8)
**Smoking statusy**			
Current	10,220 (7.9)	3,649 (7.4)	1,345 (10.4)
Previous	46,454 (35.9)	16,659 (33.6)	4,127 (31.9)
Never	72,739 (56.2)	29,286 (59.1)	7,477 (57.7)
**Alcohol consumptiony**			
Daily or almost daily	30,892 (23.9)	10,649 (21.5)	2,274 (17.6)
One to four times a week	65,185 (50.4)	25,072 (50.6)	6,108 (47.2)
One to three times a month	13,771 (10.6)	5,625 (11.3)	1,694 (13.1)
Special occasions only/never	19,565 (15.1)	8,248 (16.6)	2,873 (22.2)
**Physical activityy**			
Low	19,696 (15.2)	7,890 (15.9)	2,174 (16.8)
Medium	46,548 (36.0)	18,026 (36.3)	4,258 (32.9)
High	43,419 (33.6)	16,363 (33.0)	4,562 (35.2)
Unknown/missing	19,750 (15.3)	7,315 (14.7)	1,955 (15.1)
Mean (SD) fruit and vegetable intake, portions per day	4.82 (3.06)	4.56 (2.89)	4.34 (3.11)
Mean (SD) red and process meat intake, times per day	3.40 (2.16)	3.61 (2.19)	3.89 (2.38)
Mean (SD) sleep time	8.15 (1.01)	8.14 (1.01)	8.07 (1.10)
Multivitamin intake	19,663 (15.2)	7,521 (15.2)	1,837 (14.2)
**Artificially sweetened beverages unit/dayy**			
0	106,623 (82.4)	36,107 (72.8)	9,415 (72.7)
>0–1	16,183 (12.5)	10,820 (21.8)	2,250 (17.4)
>1	6,607 (5.1)	2,667 (5.4)	1,284 (9.9)
**Natural juices unit/day**			
0	66,095 (51.1)	20,316 (41.0)	6,586 (50.9)
>0–1	54,301 (42.0)	25,635 (51.7)	5,111 (39.5)
>1	9,017 (7.0)	3,643 (7.3)	1,252 (9.7)
Total sugar intake (mean, SD; g/day)	117.13 (46.65)	133.35 (45.34)	173.66 (65.69)
Total energy intake (mean, SD; KJ/day)	8,437.55 (2,484.16)	8,899.86 (2,376.86)	9,908.38 (3,224.16)
Aspirin use	15,555 (12.0)	5,939 (12.0)	1,590 (12.3)
NASIDs use	36,257 (28.0)	14,918 (30.1)	4,360 (33.7)
Proton pump inhibitors use	10,052 (7.8)	4,081 (8.2)	1,214 (9.4)
Metabolic syndrome	29,640 (26.7)	12,260 (28.7)	3,707 (33.2)
Central obesity	36,978 (28.6)	15,028 (30.3)	4,561 (35.3)
Hypertriglyceridemia	54,216 (44.0)	22,032 (46.7)	6,179 (50.3)
Reduced HDL	19,600 (17.6)	8,404 (19.6)	2,795 (25.0)
Elevated blood pressure	91,116 (70.4)	35,070 (70.8)	9,153 (70.7)
Hyperglycemia	17,993 (16.0)	6,643 (15.4)	1,800 (16.0)
Hyperuricemia	13,687 (11.2)	5,625 (12.0)	1,718 (14.1)

HDL, High-Density Lipoprotein; NASIDs, Non-steroidal Anti-Inflammatory Drug; SD, standard deviation.

During a median follow-up of 10.63 years, we documented 4,983 incident CKD cases. Table 2 presents associations of SSBs, ASBs, and NJs with the risk of CKD. After adjusting for sociodemographic factors, lifestyle behaviors, medication use, total sugar and energy intake, and two other beverages, all three types of beverages were associated with the risk of CKD. In the fully adjusted model, participants consuming >1 units/d of SSBs had a 45% higher risk of CKD compared with non-consumers (HR 1.45; 95% CI 1.30–1.61, *P*-trend < 0.001). Higher ASBs intake was also associated with a higher CKD risk (*P*-trend < 0.001), with HRs of 1.14 (95% CI 1.05–1.23) for >0–1 unit/d and 1.52 (95% CI 1.36–1.70) for over 1 unit/d compared to non-consumers. In contrast, participants with moderate NJ intake (>0–1 unit/d) had a 14% reduced CKD risk (HR 0.86, 95% CI 0.81–0.91) compared with non-consumers. We did not observe sufficient evidence of associations for consuming SSBs 0–1 unit/day or NJs >1 units/day compared to none (Table 2). We also found that participants consuming 0.5–1 unit/day of SSBs or ASBs were at a higher risk of CKD (HR 1.09, 95% CI 0.99–1.19 for SSBs; HR 1.31, 95% CI 1.18–1.46 for ASBs), while those consuming 0–0.5 unit/day of SSBs or ASBs did not show an increased risk compared to non-consumers (Supplementary Table 4). In substitution analyses, replacing 1 unit/d of SSBs or ASBs with an equivalent consumption of NJs was associated with a nearly 20% lower risk of CKD (HR 0.82, 95% CI 0.77–0.87 for SSBs; HR 0.80, 95% CI 0.75–0.85 for ASBs). However, substituting SSBs with ASBs did not reduce the risk of CKD (HR 1.02, 95% CI 0.97–1.08).

**Table 2:** Associations between consumption of three types of beverages and risk of chronic kidney diseases.

	**Cases/person-years**	**Hazard ratio [95% confidence interval]**		
		**Age and gender-stratified model**	**Multivariable adjusted model 1** ^†^	**Multivariable adjusted model 2** ^‡^
**Sugar-sweetened beverages**				
0 unit per day	3,273/1,368,343.4	Ref	Ref	Ref
0–1 unit per day	1,263/527,344	1.04 [0.97, 1.11]	1.02 [0.96, 1.09]	1.03 [0.96, 1.10]
>1 units per day	447/136,586.6	1.67 [1.51, 1.85]	1.49 [1.35, 1.65]	1.45 [1.30, 1.61]
*P* for trend		<0.001	<0.001	<0.001
**Artificially sweetened beverages**				
0 unit per day	3,873/1,610,144.7	Ref	Ref	Ref
0–1 unit per day	753/310,887.4	1.21 [1.12, 1.31]	1.14 [1.05, 1.23]	1.14 [1.05, 1.23]
>1 units per day	357/111,241.9	1.87 [1.67, 2.08]	1.57 [1.40, 1.75]	1.52 [1.36, 1.70]
*P* for trend		<0.001	<0.001	<0.001
**Natural juices**				
0 unit per day	2,650/982,635.8	Ref	Ref	Ref
0–1 unit per day	1,967/901,712.2	0.74 [0.70, 0.78]	0.85 [0.8, 0.91]	0.86 [0.81, 0.91]
>1 units per day	366/147,926	0.87 [0.78, 0.97]	0.99 [0.89, 1.11]	1.01 [0.90, 1.13]
*P* for trend		<0.001	0.001	0.009

† Multivariable adjusted model 1: additionally adjusted for race (white, or other), education levels (less than high school, high school or equivalent, or college or above), household income (low, medium, high, unknown, or missing), socioeconomic status (index of multiple deprivation, fifth), smoking status (never smoker, previous smoker, or current smoker), alcohol consumption (daily or almost daily, one to four times a week, one to three times a month, special occasions only or never), physical activity (low, moderate, or high), fruit and vegetable intake (≥5 portions or <5 portions), red and processed meat intake (<2.0 times per week, 2.0–2.9 times per week, 3.0–3.9 times per week, and ≥4.0 times per week), sleep time (<8, 8–9, >9 h), and medications use (aspirin, non-aspirin NSAIDs, and proton pump inhibitors use).

‡ Multivariable adjusted model 2: additionally adjusted for total sugar intake, total energy, and mutually adjusted for another two beverages.

**Figure 1:**
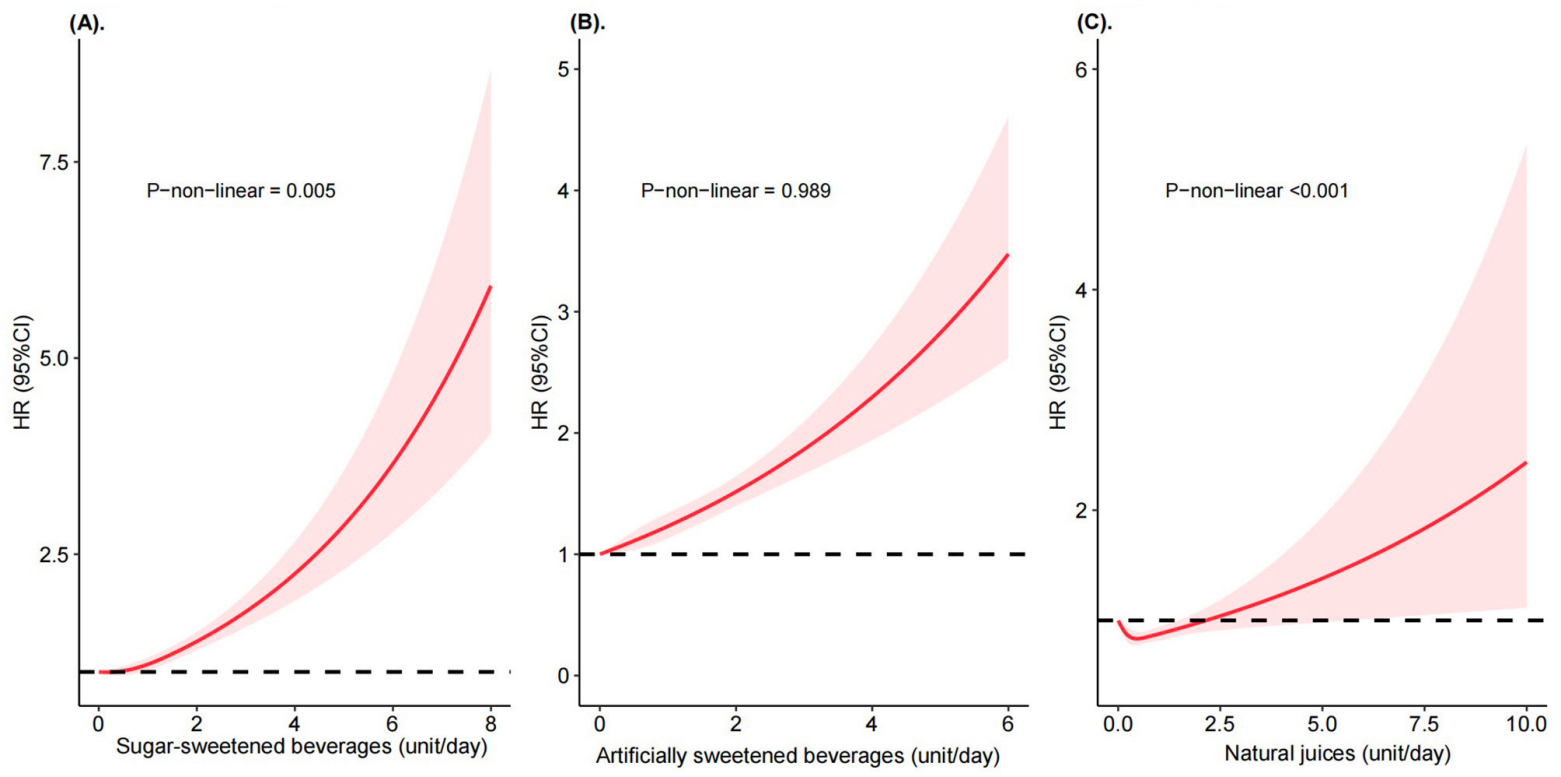
Risk of incident chronic kidney diseases according to consumption of sugar-sweetened beverages, artificially sweetened beverages, and natural juices. Restricted cubic splines of HRs were calculated from Cox proportional hazard models adjusted for sex, age, race, education levels, household income, socioeconomic status, smoking status, alcohol consumption, physical activity, fruit and vegetable intake, red and processed meat intake, sleep time, medications use (aspirin, non-aspirin NSAIDs, and proton pump inhibitors use), total sugar intake, total energy, and mutually adjusted for another two beverages. **(A)** Sugar-sweetened beverages. **(B)** Artificially sweetened beverages. **(C)** Natural juices.

In the restricted cubic splines, we observed a significant non-linear relationship between SSBs and NJs to CKD risk, but not for ASBs (*P*-non-linearity = 0.005 for SSBs, <0.001 for NJs, and 0.989 for ASBs, Figure 1 and Supplementary Figure 2). Higher SSBs intake was associated with a substantially increased CKD risk, but only if SSBs intake was above >0.5 units/day. In addition, we observed a J-shaped association between NJs and CKD risk, with the lowest risk at 0.5 unit/day.

### Mediation analyses of indirect and direct effects for beverages consumption with CKD

As shown in Supplementary Table 5, all three types of beverages were positively associated with MetS risk after adjusting for the aforementioned covariates. As expected, MetS and its individual components were associated with a higher risk of CKD (Supplementary Table 6). Table 3 presents the HRs and 95% CIs of the total effects, natural direct effects, and the natural indirect effects through MetS of the three types of beverages on CKD risk in mediation analyses. The proportion of the observed association between consuming >1 units/d of SSBs and CKD mediated by MetS was 12.5%. Similarly, the observed associations between ASBs consumption and CKD risk were also mediated by MetS, with the proportions mediated being 13.2% for consuming ASBs 0–1 unit/d and 18.0% for consuming ASBs >1 units/d. Notably, the inclusion of hyperuricemia as an extra component of MetS did not substantially change the magnitude of MetS mediated. Regarding the individual MetS component, the MetS subcomponent that accounted for the largest proportion of the mediated associations of SSBs and ASBs consumption with CKD was central obesity (9.3% for SSBs >1 units/d; 27.0% for ASBs >1 units/d; Supplementary Table 7).

**Table 3:** Adjusted direct and indirect associations of three types of beverages with CKD mediated via metabolic syndrome (MetS)^a^.

	**Sugar-sweetened beverages unit/day**			**Artificially sweetened beverages unit/day**			**Natural juices unit/day**		
	**0**	**0–1**	>**1**	**0**	**0–1**	>**1**	**0**	**0–1**	>**1**
**MetS**									
Total association^a^	Ref	1.04 [0.89, 1.21]	1.42 [1.02, 1.98]	Ref	1.44 [1.19, 1.74]	1.59 [1.26, 2.01]	Ref	0.88 [0.77, 1.00]	1.03 [0.81, 1.30]
Natural direct association		1.03 [0.88, 1.20]	1.37 [0.97, 1.94]		1.38 [1.12, 1.69]	1.48 [1.14, 1.93]		0.88 [0.77, 1.00]	1.01 [0.79, 1.29]
Natural indirect association		1.01 [1.00, 1.02]	1.04 [1.00, 1.08]		1.04 [1.01, 1.07]	1.07 [1.02, 1.13]		1.00 [1.00, 1.00]	1.01 [1.00, 1.03]
Proportion mediated (%)^b^		NA	12.5		13.2	18.0		0.6	NA
**MetS** + **hyperuricemia**									
Total association^a^	Ref	1.06 [0.90, 1.24]	1.47 [1.05, 2.07]	Ref	1.44 [1.18, 1.75]	1.58 [1.24, 2.00]	Ref	0.87 [0.76, 1.00]	1.06 [0.83, 1.34]
Natural direct association		1.04 [0.89, 1.23]	1.42 [0.99, 2.04]		1.37 [1.11, 1.70]	1.46 [1.11, 1.92]		0.87 [0.76, 1.00]	1.04 [0.81, 1.33]
Natural indirect association		1.01 [1.00, 1.02]	1.04 [1.00, 1.07]		1.05 [1.02, 1.08]	1.08 [1.02, 1.14]		1.00 [0.99, 1.00]	1.01 [1.00, 1.02]
Proportion mediated (%)^b^		NA	10.9		14.7	20.1		1.0	NA

a The HRs and 95% CI of the total effect, natural direct effect, and natural indirect effect were calculated using the VanderWeele counterfactual-framework approach (31, 32). The models were adjusted for sex, age, race, education levels, household income, socioeconomic status, smoking status, alcohol consumption, physical activity, fruit and vegetable intake, red and processed meat intake, sleep time, medications use (aspirin, non-aspirin NSAIDs, and proton pump inhibitors use), total sugar intake, total energy, and mutually adjusted for another two beverages. Total effect estimates may vary between models for different mediators due to differences in the number of missing values for each mediator and exposure-mediator interactions.

b Proportion mediated not given in cases where the null effect (i.e., 1) is contained in the 95% CI of the HR of the total effect.

In the subgroup analysis, the observed associations of ASBs and NJs with CKD did not significantly differ by sex, age, smoking status, alcohol drinking, hypertension, MetS, and predicted CKD risk (Table 4). The risk of CKD associated with higher SSB consumption did not significantly differ by household income, smoking status, alcohol consumption, hypertension, hyperglycemia, MetS, or predicted CKD risk, but appeared to be higher among women and participants under 60 years of age (*p*-interactions < 0.05). Additionally, the risk associated with higher ASB consumption appeared to be elevated in low-income participants and those with hyperglycemia (*p*-interactions < 0.05). The main results remained robust in several sensitivity analyses, such as lagging the exposure for 2 years, excluding participants with cardiovascular disease at baseline, additional adjustments for energy from beverages or kidney function at baseline, and in the competing risk analysis (Supplementary Table 8). Similar results were also found for the association between all three types of beverage consumption and acute kidney injury (AKI; Supplementary Table 9). In the fully adjusted models, both higher consumption of SSBs and ASBs were associated with an increased risk of AKI (*p*-trend < 0.001), whereas consuming 0–1 units/d of NJs was associated with an 8% lower risk of AKI (HR 0.92, 95% CI 0.87–0.98).

**Table 4:** Subgroup analyses for the associations between consumption of three types of beverages and risk of chronic kidney diseases.

	**Sugar-sweetened beverages unit/day**			**Artificially sweetened beverages unit/day**			**Natural juices unit/day**		
	**0**	**0–1**	>**1**	**0**	**0–1**	>**1**	**0**	**0–1**	>**1**
**Sex**									
Male	Ref	0.94 [0.87, 1.03]	1.40 [1.23, 1.59]	Ref	1.17 [1.06, 1.29]	1.53 [1.33, 1.77]	Ref	0.86 [0.8, 0.93]	0.96 [0.83, 1.11]
Female	Ref	1.19 [1.07, 1.32]	1.57 [1.30, 1.90]	Ref	1.1 [0.97, 1.25]	1.53 [1.29, 1.82]	Ref	0.85 [0.77, 0.94]	1.1 [0.9, 1.34]
*P* for interaction	0.006			0.906			0.505		
**Age**									
37–60 years	Ref	1.12 [1.00, 1.25]	1.52 [1.29, 1.78]	Ref	1.17 [1.03, 1.33]	1.56 [1.32, 1.83]	Ref	0.89 [0.8, 0.98]	1.01 [0.84, 1.23]
≥60 years	Ref	0.98 [0.90, 1.07]	1.40 [1.22, 1.61]	Ref	1.12 [1.01, 1.23]	1.5 [1.29, 1.74]	Ref	0.84 [0.78, 0.91]	1 [0.86, 1.15]
*P* for interaction	0.025			0.442			0.988		
**Household income**									
Low	Ref	1.04 [0.91, 1.19]	1.42 [1.16, 1.73]	Ref	1.22 [1.04, 1.43]	1.88 [1.53, 2.31]	Ref	0.87 [0.77, 0.99]	0.98 [0.77, 1.26]
Medium	Ref	1.01 [0.92, 1.12]	1.46 [1.25, 1.71]	Ref	1.18 [1.05, 1.32]	1.50 [1.28, 1.77]	Ref	0.87 [0.80, 0.95]	1.00 [0.84, 1.18]
High	Ref	1.01 [0.86, 1.2]	1.64 [1.25, 2.16]	Ref	1.07 [0.88, 1.30]	1.14 [0.83, 1.57]	Ref	0.85 [0.73, 0.99]	1.08 [0.84, 1.39]
*P* for interaction	0.773			0.004			0.616		
**Never smoker**									
No	Ref	0.97 [0.89, 1.07]	1.45 [1.26, 1.67]	Ref	1.1 [0.99, 1.22]	1.5 [1.3, 1.74]	Ref	0.87 [0.8, 0.94]	0.96 [0.81, 1.12]
Yes	Ref	1.09 [0.99, 1.2]	1.47 [1.26, 1.73]	Ref	1.16 [1.03, 1.31]	1.51 [1.27, 1.8]	Ref	0.84 [0.77, 0.92]	1.05 [0.89, 1.23]
*P* for interaction	0.228			0.712			0.408		
**Never drinker**									
No	Ref	1.01 [0.93, 1.09]	1.41 [1.24, 1.6]	Ref	1.12 [1.03, 1.23]	1.51 [1.32, 1.72]	Ref	0.81 [0.76, 0.87]	0.97 [0.85, 1.11]
Yes	Ref	1.08 [0.94, 1.25]	1.58 [1.3, 1.93]	Ref	1.18 [1, 1.38]	1.61 [1.32, 1.96]	Ref	0.97 [0.85, 1.11]	1.07 [0.84, 1.35]
*P* for interaction	0.284			0.461			0.151		
**Hypertension**									
No	Ref	1.03 [0.86, 1.23]	1.74 [1.33, 2.29]	Ref	1.13 [0.91, 1.4]	1.29 [0.93, 1.80]	Ref	0.86 [0.73, 1.02]	0.99 [0.72, 1.35]
Yes	Ref	1.04 [0.97, 1.12]	1.45 [1.29, 1.63]	Ref	1.1 [1.01, 1.19]	1.47 [1.31, 1.65]	Ref	0.86 [0.81, 0.92]	1.02 [0.9, 1.15]
*P* for interaction	0.354			0.69			0.997		
**Hyperglycemia**									
No	Ref	1.08 [0.99, 1.17]	1.41 [1.23, 1.63]	Ref	1.04 [0.94, 1.16]	1.23 [1.05, 1.45]	Ref	0.85 [0.79, 0.92]	0.93 [0.8, 1.08]
Yes	Ref	0.91 [0.8, 1.04]	1.51 [1.24, 1.83]	Ref	1.21 [1.05, 1.39]	1.64 [1.38, 1.94]	Ref	0.87 [0.77, 0.98]	1.28 [1.04, 1.58]
*P* for interaction	0.078			0.002			0.123		
**Metabolic syndrome**									
No	Ref	1.08 [0.97, 1.19]	1.36 [1.14, 1.62]	Ref	1.12 [0.99, 1.28]	1.32 [1.07, 1.64]	Ref	0.8 [0.73, 0.87]	0.91 [0.77, 1.09]
Yes	Ref	0.94 [0.85, 1.04]	1.39 [1.19, 1.63]	Ref	1.14 [1.01, 1.27]	1.46 [1.26, 1.69]	Ref	0.9 [0.82, 0.98]	1.11 [0.93, 1.32]
*P* for interaction	0.121			0.402			0.561		
**The predicted 5-year CKD risk**									
Low	Ref	1.15 [0.94, 1.39]	1.67 [1.29, 2.18]	Ref	1.32 [1.07, 1.64]	1.61 [1.22, 2.12]	Ref	0.85 [0.71, 1.02]	0.97 [0.70, 1.35]
Medium	Ref	1.03 [0.91, 1.17]	1.53 [1.26, 1.86]	Ref	1.14 [0.98, 1.32]	1.58 [1.28, 1.94]	Ref	0.84 [0.75, 0.94]	1.13 [0.92, 1.39]
High	Ref	1.00 [0.92, 1.09]	1.35 [1.16, 1.56]	Ref	1.09 [0.98, 1.21]	1.45 [1.25, 1.68]	Ref	0.87 [0.80, 0.94]	0.95 [0.82, 1.11]
*P* for interaction	0.1035			0.304			0.601		

Estimated effects were based on the fully adjusted model (see the footnote in Table 2). The predicted 5-year CKD risk were calculated by previously developed 5-year risk prediction equations for CKD and then categorized into three groups by tertiles (low, medium, and high).

## Discussion

In this prospective analysis of over 0.19 million participants, we found that SSB intake of >1 units/day or ASB intake of >0 units/day was each associated with a higher risk of CKD, whereas moderate NJs consumption (0–1 unit/day) was associated with a lower risk. These associations were robust across major subgroups and in sensitivity analyses. Moreover, we found that over 10% of the total association of CKD with SSBs or ASBs consumption was partly mediated through MetS. Regarding individual components of MetS, central obesity mediated the highest proportion of the observed associations of CKD with SSBs or ASBs consumption. These findings highlight the importance of controlling excessive SSB or ASB consumption for CKD prevention. Furthermore, the findings identified the MetS processes as a potentially influential pathway linking SSBs or ASBs consumption to CKD risk.

Previous studies regarding the associations between sugary beverages and CKD have yielded inconsistent results (15–22). Consistent with our findings, a prospective cohort study based on Jackson Heart Study found that higher consumption of SSBs was associated with an increased risk of CKD (OR 1.18, 95% CI, 1.00–1.39) (19). Another study based on the Nurses' Health Study (NHS) indicated that consuming artificially sweetened soda was associated with nearly a 2-fold higher risk of eGFR decline ≥30% compared with consuming <1 servings per month over the 11 years of follow-up (OR 2.02, 95% CI 1.36–3.01), but whereas the association was non-significant for sugar soda (OR for ≥ 1 servings per day vs. <1 servings per month = 1.56, 95% CI 1.84–1.91) (21). In contrast, a cohort study based on the Atherosclerosis Risk in Communities (ARIC) Study showed no association between sweetened beverages and CKD risk (*P*-trend = 0.30) (22), while a prospective analysis based on the Tehran Lipid and Glucose Study (TLGS) showed a negative association (quintile 5 vs. quintile 1, OR 0.54, 95% CI 0.32–0.94) (18). A recent meta-analysis revealed no association between higher consumption SSBs or ASBs and CKD risk, but reported a higher CKD risk in participants consuming SSBs or ASBs over seven servings per week (15). Given the differences in measurements of beverages, sample size, and cultural background across studies, our findings not only contribute to the current literature in a UK context but also provide further evidence supporting the potential association of SSBs and ASBs with a higher risk of CKD.

As a potential alternative drink for SSBs and ASBs, we found that moderate intake of NJs was associated with a lower risk of CKD, which was in contrast with a previous study that found no association between NJs and CKD risk (19). Furthermore, our substitution analyses showed replacing 1 unit/d of SSBs or ASB with an equivalent consumption of NJs was associated with a nearly 20% lower risk of CKD. Given that the moderate intake of NJs has been linked to a lower risk of central obesity (33), cardiometabolic multimorbidity (34, 35), and dementia (24), our findings suggest that moderate consumption of pure fruit/vegetable juices may be a healthier alternative to SSBs and ASBs for CKD prevention. However, the results from restricted cubic splines suggested participants taking NJ >6 units/d had a higher CKD risk compared those who did not drink any. Since excessive NJs consumption has been linked to a higher risk of weight gain, type 2 diabetes, and increased all-cause mortality (12, 36, 37), further research on fruit juice is warranted to define the optimal intake level.

To our knowledge, this is the first study to investigate that the association between sugar beverages and increased CKD risk may be partly mediated via MetS. Previous studies focused predominantly on the main effect of sugar beverages on CKD risk, neglecting the possibility of multiple pathways and processes (e.g., MetS) linking them (18–22). Our findings, indicating that MetS and its components partially mediate the associations of sugary beverages with CKD risk, offer insights into the mechanisms linking these beverages to CKD development. Notably, higher consumption of SSBs and ASBs has consistently been associated with an elevated risk of obesity, hypertension, diabetes, and MetS (12, 38–40), and MetS and its components were well-established risk factors for CKD (41). This confirmed the observed associations in the study were biologically plausible. Moreover, our findings extend previous research on individual MetS components, such as obesity, which has been shown to mediate the association between sugary beverages and diabetes and cardiovascular disease (42–44). However, given the relatively small mediation proportion of MetS and its individual components, the effect of SSBs and ASBs on CKD is likely explained largely through mechanisms other than MetS, such as chronic low-grade inflammation (20, 45). Further research is needed to explore these mechanisms comprehensively.

The strengths of the present study included data from a well-characterized prospective cohort with large sample size and long-term follow-up, as well as detailed information on sugar beverages, MetS and incident of CKD. The sensitivity and subgroup analyses have confirmed the validity of the findings. In addition, our mediation analyses based on the counterfactual framework provided clues for the underlying mechanisms connecting sugar beverage consumption to CKD risk.

This research has certain limitations. Firstly, sugar beverage consumption was assessed through 24-h diet recall questionnaires, which might be susceptible to recall or reporting biases. Secondly, despite adjusting for several potential confounding factors, the potential for residual confounding from unknown or unmeasured factors cannot be completely ruled out. Thirdly, while efforts were made to minimize reverse causality by lagging the exposure for 2 years, its possibility still exists. Fourthly, as the study participants were primarily from the UK Biobank, and the majority were of white ethnicity, the generalizability of the findings to other racial groups requires further investigation. Fifthly, the observational nature of this study limited our ability to establish causal relationships. Lastly, mediation analysis assumes temporal causation between sugary beverage intake and MetS development, despite both the sugary beverages intake and MetS development were measured at the same time in the UK Biobank. More research with longitudinal data is needed to validated our findings.

## Conclusion

This large cohort study found that higher intake of SSBs and ASBs was associated with an increased CKD risk, whereas moderate intake of NJs have protective effect on CKD development. The observed associations partially mediated by MetS. Although the causal relationship cannot be established, our results emphasize the critical importance of limiting the consumption of SSBs or ASBs for CKD prevention. Further research is needed to confirm our findings and explore the optimal intake level for the natural juices.

## Data Availability

The datasets presented in this study can be found in online repositories. The names of the repository/repositories and accession number(s) can be found at: UK Biobank. Available at: https://www.ukbiobank.ac.uk/ (accessed June 01, 2020).
