# Supplementary material for: Sugary beverages intake and risk of chronic kidney disease: the mediating role of metabolic syndrome
Source: Front Nutr. 2024 Nov 26;11:1401081. doi: 10.3389/fnut.2024.1401081 (PMC11628267; doi:10.3389/fnut.2024.1401081)
Supplement: Supplementary file 1 [file Data_Sheet_1.docx]

Table S1. The definition of variables in the study

| **Variables** | **Data-Field in UK Biobank** | **Description** | **Definition of variables** |
| --- | --- | --- | --- |
| **Outcome** |  |  |  |
| CKD incidence | 132032 | Date N18 first reported (chronic renal failure) | ICD 10 code |
| **Exposures** |  |  |  |
| Sugar-sweetened beverages | 100170 | Fizzy drink intake | Participants were categorized into three groups based on consumption of each beverage as follows: 0, 0–1, >1 unit per day, respectively |
|  | 100180 | Squash intake |  |
| Artificially-sweetened beverages | 100160 | Low calorie drink intake |  |
| Natural juices | 100190 | Orange juice intake |  |
|  | 100200 | Grapefruit juice intake |  |
|  | 100210 | Pure fruit/vegetable juice intake |  |
| **Metabolic syndrome components** | |  |  |
| Central obesity | 48 | Waist circumference | Waist circumference over 88 cm in women or 102 cm in men |
| Hyperglycemia | 30740 | Glucose | Fasting blood sugar over 100 mg/dl or previously diagnosed type 2 diabetes or taking medication for diabetes |
|  | 30750 | Glycated haemoglobin (HbA1c) |  |
|  | 2443 | Diabetes diagnosed by doctor |  |
|  | 41270 | Diagnoses - ICD10 |  |
|  | 41271 | Diagnoses - ICD9 |  |
|  | 20002 | Non-cancer illness code, self-reported |  |
|  | 6177 | Medication for cholesterol, blood pressure or diabetes |  |
|  | 6153 | Medication for cholesterol, blood pressure, diabetes, or take exogenous hormones |  |
| Elevated blood pressure | 94 | Diastolic blood pressure, manual reading | Systolic pressure ≥ 130 mm Hg, diastolic ≥85 mm Hg, or antihypertensive treatment for previously diagnosed hypertension |
|  | 93 | Systolic blood pressure, manual reading |  |
|  | 6150 | Vascular/heart problems diagnosed by doctor |  |
| Hypertriglyceridemia | 30870 | Triglycerides | Fasting triglyceride (TG) level over 150 mg/dl or currently on medications for hypertriglyceridemia. |
| Reduced HDL | 30760 | HDL cholesterol | Fasting high-density lipoprotein (HDL) cholesterol level less than 40 mg/dl (men) or 50 mg/dl (women), or specific treatment for previously detected reduced HDL cholesterol. |
| Hyperuricemia | 30880 | Urate | Serum urate over 7 mg/dL in men or 6 mg/dL in women |
| Metabolic syndrome |  |  | 3 or more metrics met: central obesity, hypertriglyceridemia, reduced high-density lipoprotein cholesterol, elevated blood pressure and hyperglycemia |
| MetS+ Hyperuricemia | | | 3 or more metrics met: central obesity, hypertriglyceridemia, reduced high-density lipoprotein cholesterol, elevated blood pressure and hyperglycemia, as well as hyperuricemia |
| **Covariates** |  |  |  |
| Age | 21022 | Age at recruitment | Age was categorized into three groups (≤50, 50-60, and >60 years). |
| Sex | 31 | Sex | Data-Coding in the UK biobank: male; female |
| Ethnicity | 21000 | Ethnic background | The white category comprised British, Irish, and other white background. Other category including other ethnic group |
| Index of multiple deprivation | 26410 | Index of Multiple Deprivation (England) | Deprivation was categorized into origin specific quintiles among England, Scotland, and Wales separately |
|  | 26427 | Index of Multiple Deprivation (Scotland) |  |
|  | 26426 | Index of Multiple Deprivation (Wales) |  |
| Education | 6138 | Qualifications | Data-Coding in the UK biobank: college or university degree; A levels, AS levels, or equivalent; O levels, GCSEs, or equivalent; CSEs or equivalent; NVQ, HND, HNC, or equivalent; other professional qualifications; none of the above. We recoded into three groups: College or above (college or university degree), Less than high school (none of the above) and high school or equivalent (other options) |
| Income | [738](https://biobank.ndph.ox.ac.uk/showcase/field.cgi?id=738) | [Average total household income before tax](https://biobank.ndph.ox.ac.uk/showcase/field.cgi?id=738) | Data-Coding in the UK biobank: "<£18 000", "£18 000-£30 999"," £31 000-£51 999","£52 000-£100 000", ">£100 000", "do not know", or "Prefer not to answer". We recoded into three groups: low ( <£18 000), medium (£18 000-£51 999), and high (≥£52 000) family income level, and created an “unknown/missing” response category for "prefer not to answer’, or missing data |
| Smoking status | 20116 | Smoking status |  |
| Alcohol consumption | 1558 | Alcohol intake frequency. | Data-Coding in the UK biobank: "Daily or almost daily", "Three or four times a week", "Once or twice a week", "One to three times a month" , "Special occasions only"," Never", "Prefer not to answer". We combined "Three or four times a week" and "Once or twice a week" into a group "One to four times a week", and "Special occasions only" and " Never" into a group "Special occasions only/Never" |
| Physical activity | 22032 | IPAQ activity group | Data-Coding in the UK biobank: Low, moderate, high. An “unknown/missing” response category was created missing data |
|  | 22040 | Summed MET minutes per week for all activity |  |
| Fruit and vegetable intake | 1289 | Cooked vegetable intake | Based on the UK guidelines that a portion of vegetables is three heaped tablespoons, each piece of fruit counted as one portion and three heaped tablespoons of vegetables counted as one portion. We categorized participants into those who consumed <5 portions per day and those who consumed ≥5 based on the UK’s current guidelines. |
|  | 1299 | Salad / raw vegetable intake |  |
|  | 1309 | Fresh fruit intake |  |
|  | 1319 | Dried fruit intake |  |
| Red and process meat intake | 1349 | Processed meat intake | Data-Coding in the UK biobank: ‘never, ‘less than once a week’, ‘once a week’, ‘2-4 times a week’, ‘5-6 times a week’, ‘once or more daily’, ‘do not know’, ‘prefer not to answer’. For processed meat, the top three frequencies were combined to create four categories: never, <1.0 time per week, 1.0 time per week, and ≥2.0 times per week’. We summed the frequencies for beef, pork, and lamb/mutton to calculate the red meat consumption, using the following coding: ‘Never’ = 0, ‘Less than once a week’ = 0.5, ‘Once a week’ = 1, ‘2-4 times a week’ = 3, ‘5-6 times a week’ = 5.5, ‘Once or more daily’ = 7. The categories for red meat consumption were: <1 time per week, 1.0-1.9 times per week, 2.0-2.9 times per week, and ≥3.0 times per week. For red and processed meat, we summed the frequencies for red and processed meat. The categories for red and processed meat consumption were: < 2.0 times per week, 2.0-2.9 times per week, 3.0-3.9 times per week, and ≥4.0 times per week.(**ref: Bradbury KE., IJE, 49(1), 246-258.**) |
|  | 1369 | Beef intake |  |
|  | 1379 | Lamb/mutton intake |  |
|  | 1389 | Pork intake |  |
| Sleep time | 1160 | Sleep duration | Participants were categorized into three groups based on sleep duration as follows: <8 hours, 8-9hours, ＞9 hours, respectively |
| Multivitamin intake | 6155 | Vitamin and mineral supplements | Any use of Vitamin A, Vitamin B, Vitamin C, Vitamin D, Vitamin E |
| Regular use of Aspirin/NSAIDs/PPIs | 6154 | Medication for pain relief, constipation, heartburn | Any use of aspirin, ibuprofen, paracetamol, omeprazole and so on |
|  | 2492 | Taking other prescription medications |  |
| Total sugar intake | 26012 | Free sugar | Total sugar intake was treated as continuous variable |
| Total energy intake | 26002 | Energy | Total energy intake was treated continuous variable |

| Table S2. Baseline characteristics of participants by consumption of artificially sweetened beverages | | | |
| --- | --- | --- | --- |
|  | Artificially sweetened beverages, unit/day | | |
|  | 0 | 0–1 | >1 |
| Number of participants | 152145 | 29253 | 10558 |
| Mean (SD) Age, years | 56.68 (7.87) | 54.98 (7.93) | 53.40 (7.98) |
| Male | 70750 (46.5) | 12339 (42.2) | 4401 (41.7) |
| N (%) White | 145377 (95.6) | 28023 (95.8) | 10163 (96.3) |
| Mean (SD) index of multiple deprivation | 15.19 (12.18) | 15.73 (12.53) | 17.34 (13.60) |
| Education |  |  |  |
| Less than high school | 13376 (8.8) | 2508 (8.6) | 913 (8.6) |
| High school or equivalent | 56532 (37.2) | 12024 (41.1) | 4585 (43.4) |
| College or above | 82237 (54.1) | 14721 (50.3) | 5060 (47.9) |
| Household income |  |  |  |
| Low | 20927 (13.8) | 3665 (12.5) | 1398 (13.2) |
| Medium | 72082 (47.4) | 13684 (46.8) | 4990 (47.3) |
| High | 43346 (28.5) | 9052 (30.9) | 3170 (30.0) |
| Unknown/missing | 15790 (10.4) | 2852 (9.7) | 1000 (9.5) |
| Smoking status |  |  |  |
| Current | 12116 ( 8.0) | 2146 (7.3) | 952 ( 9.0) |
| Previous | 52874 (34.8) | 10487 (35.8) | 3879 (36.7) |
| Never | 87155 (57.3) | 16620 (56.8) | 5727 (54.2) |
| Alcohol consumption |  |  |  |
| Daily or almost daily | 36042 (23.7) | 5962 (20.4) | 1811 (17.2) |
| One to four times a week | 76491 (50.3) | 14932 (51.0) | 4942 (46.8) |
| One to three times a month | 16074 (10.6) | 3526 (12.1) | 1490 (14.1) |
| Special occasions only/Never | 23538 (15.5) | 4833 (16.5) | 2315 (21.9) |
| Physical activity |  |  |  |
| Low | 23103 (15.2) | 4773 (16.3) | 1884 (17.8) |
| Medium | 54787 (36.0) | 10530 (36.0) | 3515 (33.3) |
| High | 51022 (33.5) | 9798 (33.5) | 3524 (33.4) |
| Unknown/missing | 23233 (15.3) | 4152 (14.2) | 1635 (15.5) |
| Mean (SD) Fruit and vegetable intake, portions per day | 4.74 (3.04) | 4.63 (2.94) | 4.57 (3.03) |
| Mean (SD) red and process meat intake, times per day | 3.46 (2.18) | 3.55 (2.15) | 3.75 (2.31) |
| Mean (SD) Sleep time | 8.15 (1.01) | 8.14 (1.02) | 8.10 (1.10) |
| Multivitamin intake | 23302 (15.3) | 4226 (14.4) | 1493 (14.1) |
| Sugar-sweetened beverages, unit/day |  |  |  |
| 0 | 106623 (70.1) | 16183 (55.3) | 6607 (62.6) |
| >0–1 | 36107 (23.7) | 10820 (37.0) | 2667 (25.3) |
| >1 | 9415 ( 6.2) | 2250 ( 7.7) | 1284 (12.2) |
| Natural juices, unit/day |  |  |  |
| 0 | 72902 (47.9) | 13925 (47.6) | 6170 (58.4) |
| >0–1 | 67744 (44.5) | 13543 (46.3) | 3760 (35.6) |
| >1 | 11499 ( 7.6) | 1785 ( 6.1) | 628 ( 5.9) |
| Total sugar intake (mean, SD) (g/day) | 125.48 (49.55) | 123.46 (48.94) | 124.84 (59.88) |
| Total energy intake (mean, SD) (KJ/day) | 8669.73 (2525.56) | 8575.83 (2495.33) | 8684.06 (2919.79) |
| Aspirin use | 17829 (11.7) | 3676 (12.6) | 1579 (15.0) |
| NASIDs use | 42042 (27.6) | 9498 (32.5) | 3995 (37.8) |
| Proton pump inhibitors use | 11657 ( 7.7) | 2613 ( 8.9) | 1077 (10.2) |
| Metabolic syndrome | 33583 (25.7) | 8335 (33.2) | 3689 (40.7) |
| Central obesity | 40545 (26.7) | 10952 (37.5) | 5070 (48.1) |
| Hypertriglyceridemia | 64137 (44.3) | 13211 (47.5) | 5079 (50.5) |
| Reduced HDL | 22593 (17.2) | 5638 (22.4) | 2568 (28.2) |
| Elevated blood pressure | 106851 (70.3) | 20858 (71.3) | 7630 (72.3) |
| Hyperglycemia | 19871 (15.1) | 4552 (17.9) | 2013 (21.8) |
| Hyperuricemia | 15869 (11.1) | 3577 (13.0) | 1584 (16.0) |

HDL, High-Density Lipoprotein; NASIDs, Nonsteroidal Anti-Inflammatory Drug; SD, standard deviation.

| Table S3. Baseline characteristics of participants by consumption of natural juices | | | |
| --- | --- | --- | --- |
|  | Natural juices, unit/day | | |
|  | 0 | 0–1 | >1 |
| Number of participants | 92997 | 85047 | 13912 |
| Mean (SD) Age, years | 55.94 (7.97) | 56.63 (7.88) | 55.84 (8.00) |
| Male | 40231 (43.3) | 39929 (46.9) | 7330 (52.7) |
| N (%) White | 88568 (95.2) | 82013 (96.4) | 12982 (93.3) |
| Mean (SD) index of multiple deprivation | 16.39 (12.97) | 14.36 (11.53) | 15.11 (12.04) |
| Education |  |  |  |
| Less than high school | 10228 (11.0) | 5766 ( 6.8) | 803 ( 5.8) |
| High school or equivalent | 38211 (41.1) | 30517 (35.9) | 4413 (31.7) |
| College or above | 44558 (47.9) | 48764 (57.3) | 8696 (62.5) |
| Household income |  |  |  |
| Low | 14313 (15.4) | 9997 (11.8) | 1680 (12.1) |
| Medium | 44181 (47.5) | 40424 (47.5) | 6151 (44.2) |
| High | 24291 (26.1) | 26445 (31.1) | 4832 (34.7) |
| Unknown/missing | 10212 (11.0) | 8181 ( 9.6) | 1249 ( 9.0) |
| Smoking status |  |  |  |
| Current | 8819 (9.5) | 5372 (6.3) | 1023 ( 7.4) |
| Previous | 33757 (36.3) | 29108 (34.2) | 4375 (31.4) |
| Never | 50421 (54.2) | 50567 (59.5) | 8514 (61.2) |
| Alcohol consumption |  |  |  |
| Daily or almost daily | 19551 (21.0) | 20932 (24.6) | 3332 (24.0) |
| One to four times a week | 45512 (48.9) | 44116 (51.9) | 6737 (48.4) |
| One to three times a month | 11089 (11.9) | 8599 (10.1) | 1402 (10.1) |
| Special occasions only/Never | 16845 (18.1) | 11400 (13.4) | 2441 (17.5) |
| Physical activity |  |  |  |
| Low | 14867 (16.0) | 12839 (15.1) | 2054 (14.8) |
| Medium | 32065 (34.5) | 31748 (37.3) | 5019 (36.1) |
| High | 30950 (33.3) | 28383 (33.4) | 5011 (36.0) |
| Unknown/missing | 15115 (16.3) | 12077 (14.2) | 1828 (13.1) |
| Mean (SD) Fruit and vegetable intake, portions per day | 4.58 (3.04) | 4.78 (2.88) | 5.28 (3.64) |
| Mean (SD) red and process meat intake, times per day | 3.50 (2.22) | 3.48 (2.13) | 3.45 (2.26) |
| Mean (SD) Sleep time | 8.13 (1.05) | 8.17 (0.97) | 8.11 (1.01) |
| Multivitamin intake | 13381 (14.4) | 13189 (15.5) | 2451 (17.6) |
| Sugar-sweetened beverages, unit/day |  |  |  |
| 0 | 66095 (71.1) | 54301 (63.8) | 9017 (64.8) |
| >0–1 | 20316 (21.8) | 25635 (30.1) | 3643 (26.2) |
| >1 | 6586 ( 7.1) | 5111 ( 6.0) | 1252 ( 9.0) |
| Artificially sweetened beverages, unit/day |  |  |  |
| 0 | 72902 (78.4) | 67744 (79.7) | 11499 (82.7) |
| >0–1 | 13925 (15.0) | 13543 15.9) | 1785 (12.8) |
| >1 | 6170 (6.6) | 3760 (4.4) | 628 ( 4.5) |
| Total sugar intake (mean, SD) (g/day) | 113.75 (48.80) | 130.84 (44.55) | 166.43 (61.61) |
| Total energy intake (mean, SD) (KJ/day) | 8349.48 (2593.11) | 8829.62 (2356.79) | 9646.54 (2938.92) |
| Aspirin use | 11294 (12.1) | 10073 (11.8) | 1717 (12.3) |
| NASIDs use | 28504 (30.7) | 23344 (27.4) | 3687 (26.5) |
| Proton pump inhibitors use | 8262 ( 8.9) | 6142 ( 7.2) | 943 ( 6.8) |
| Metabolic syndrome | 23137 (29.0) | 19210 (26.2) | 3260 (27.3) |
| Central obesity | 29738 (32.0) | 23017 (27.1) | 3812 (27.4) |
| Hypertriglyceridemia | 39924 (45.1) | 36350 (44.9) | 6153 (46.6) |
| Reduced HDL | 15889 (19.8) | 12626 (17.2) | 2284 (19.1) |
| Elevated blood pressure | 65043 (70.0) | 60401 (71.0) | 9895 (71.2) |
| Hyperglycemia | 13359 (16.6) | 11256 (15.3) | 1821 (15.1) |
| Hyperuricemia | 10587 (12.1) | 8917 (11.1) | 1526 (11.6) |

HDL, High-Density Lipoprotein; NASIDs, Nonsteroidal Anti-Inflammatory Drug; SD, standard deviation.

| Table S4. Associations between consumption of three types of beverages and risk of chronic kidney diseases | | |
| --- | --- | --- |
|  | Cases/ Person-years | Hazard Ratio [95% Confidence Interval] |
| **Sugar-sweetened beverages** |  |  |
| 0 unit per day | 3273/1368343.4 | 1.00[Reference] |
| 0–0.5 unit per day | 718/313708.3 | 1.00[0.92, 1.08] |
| 0.5–1 unit per day | 545/213635.8 | 1.09[0.99, 1.19] |
| >1 units per day | 447/136586.6 | 1.45[1.31, 1.62] |
| **Artificially sweetened beverages** | |  |
| 0 unit per day | 3873/1610144.7 | 1.00[Reference] |
| 0–0.5 unit per day | 365/169443.0 | 0.99[0.89, 1.11] |
| 0.5–1 unit per day | 368/141444.5 | 1.31[1.18, 1.46] |
| >1 units per day | 357/111241.9 | 1.52[1.36, 1.70] |
| **Natural juices** |  |  |
| 0 unit per day | 2650/982635.8 | 1.00[Reference] |
| 0–0.5 unit per day | 1000/452075.2 | 0.87[0.81, 0.94] |
| 0.5–1 unit per day | 967/449637.0 | 0.86[0.80, 0.93] |
| >1 units per day | 366/147926 | 1.01[0.90, 1.14] |

Estimated effects were based on the fully adjusted model (see the footnote in Table 2).

| Table S5. Association of three types of beverages intake with metabolic syndrome and its subcomponents | | | | | | | | | | | |
| --- | --- | --- | --- | --- | --- | --- | --- | --- | --- | --- | --- |
|  | Sugar-sweetened beverages unit/day | | |  | Artificially sweetened beverages unit/day | | |  | Natural juices unit/day | | |
|  | 0 | 0–1 | >1 |  | 0 | 0–1 | >1 |  | 0 | 0–1 | >1 |
| Metabolic syndrome | Ref | 1.21[1.15, 1.28] | 1.58[1.38, 1.79] |  | Ref | 1.51[1.41, 1.62] | 1.90[1.74, 2.07] |  | Ref | 0.99[0.95, 1.03] | 1.16[1.06, 1.26] |
| MetS + Hyperuricemia | Ref | 1.21[1.15, 1.27] | 1.61[1.42, 1.83] |  | Ref | 1.52[1.42, 1.63] | 1.86[1.71, 2.02] |  | Ref | 0.98[0.94, 1.03] | 1.15[1.05, 1.24] |
| Central obesity | Ref | 1.20[1.14, 1.26] | 1.70[1.50, 1.93] |  | Ref | 1.65[1.55, 1.76] | 2.10[1.93, 2.28] |  | Ref | 0.94[0.90, 0.99] | 1.22[1.12, 1.32] |
| Hypertriglyceridemia | Ref | 1.17[1.11, 1.23] | 1.36[1.20, 1.54] |  | Ref | 1.24[1.16, 1.33] | 1.39[1.28, 1.51] |  | Ref | 1.05[1.01, 1.09] | 1.11[1.03, 1.20] |
| Reduced HDL | Ref | 1.15[1.08, 1.21] | 1.46[1.27, 1.67] |  | Ref | 1.32[1.22, 1.42] | 1.49[1.36, 1.63] |  | Ref | 1.00[0.95, 1.05] | 1.03[0.93, 1.13] |
| Elevated blood pressure | Ref | 1.06[1.00, 1.11] | 1.13[0.99, 1.30] |  | Ref | 1.16[1.08, 1.24] | 1.27[1.16, 1.40] |  | Ref | 1.05[1.01, 1.10] | 1.07[0.98, 1.17] |
| Hyperglycemia | Ref | 1.03[0.97, 1.10] | 1.28[1.09, 1.50] |  | Ref | 1.38[1.28, 1.50] | 1.67[1.52, 1.84] |  | Ref | 0.98[0.93, 1.03] | 1.13[1.02, 1.25] |
| Hyperuricemia | Ref | 1.16[1.08, 1.25] | 1.57[1.33, 1.85] |  | Ref | 1.25[1.14, 1.37] | 1.48[1.32, 1.65] |  | Ref | 0.94[0.88, 0.99] | 1.00[0.89, 1.13] |
|  |  |  |  |  |  |  |  |  |  |  |  |

Estimated effects (ORs) were calculated based on the fully adjusted logistic regression models, with adjustment for adjusted for sex, age, race, education levels, household income, socioeconomic status, smoking status, alcohol consumption, physical activity, fruit and vegetable intake, red and processed meat intake, sleep time (<8 hours, 8-9hours, ＞9 hours, medications use (aspirin, non-aspirin NSAIDs, and proton pump inhibitors use), total sugar intake, total energy, and another two beverages.

| Table S6. Associations of with metabolic syndrome and its subcomponents with chronic kidney diseases | |
| --- | --- |
|  | Hazard Ratio [95% Confidence Interval] |
| Metabolic syndrome | 1.65[1.48, 1.83] |
| MetS + Hyperuricemia | 1.69[1.51, 1.88] |
| Central obesity | 1.77[1.59, 1.97] |
| Hypertriglyceridemia | 1.23[1.10, 1.37] |
| Reduced HDL | 1.23[1.09, 1.39] |
| Elevated blood pressure | 1.46[1.26, 1.70] |
| Hyperglycemia | 1.64[1.47, 1.84] |
| Hyperuricemia | 1.54[1.36, 1.74] |

Estimated effects were based on the fully adjusted model, with adjustment for adjusted for sex, age, race, education levels, household income, socioeconomic status, smoking status, alcohol consumption, physical activity, fruit and vegetable intake, red and processed meat intake, sleep time (<8 hours, 8-9hours, ＞9 hours, medications use (aspirin, non-aspirin NSAIDs, and proton pump inhibitors use), total sugar intake, total energy, and three types of beverages.

| Table S7. Adjusted direct and indirect associations of three types of beverages with CKD mediated via subcomponents of metabolic syndrome ^a^ | | | | | | | | | | | |
| --- | --- | --- | --- | --- | --- | --- | --- | --- | --- | --- | --- |
|  | Sugar-sweetened beverages unit/day | | |  | Artificially sweetened beverages unit/day | | |  | Natural juices unit/day | | |
|  | 0 | 0–1 | >1 |  | 0 | 0–1 | >1 |  | 0 | 0–1 | >1 |
| **Central obesity** |  |  |  |  |  |  |  |  |  |  |  |
| Total association ^a^ | Ref | 1.05[0.91, 1.21] | 1.53[1.13, 2.07] |  | Ref | 1.49[1.26, 1.77] | 1.63[1.32, 2.01] |  | Ref | 0.88[0.78, 0.99] | 1.10[0.89, 1.37] |
| Natural direct association |  | 1.04[0.90, 1.19] | 1.48[1.07, 2.04] |  |  | 1.41[1.17, 1.69] | 1.46[1.15, 1.86] |  |  | 0.88[0.78, 1.00] | 1.09[0.87, 1.36] |
| Natural indirect association |  | 1.01[1.00, 1.03] | 1.03[1.00, 1.07] |  |  | 1.06[1.02, 1.10] | 1.12[1.04, 1.20] |  |  | 0.99[0.99, 1.00] | 1.01[1.00, 1.03] |
| Proportion mediated (%) ^b^ |  | NA | 9.3 |  |  | 17.8 | 27.0 |  |  | 4.3 | NA |
| **Hypertriglyceridemia** |  |  |  |  |  |  |  |  |  |  |  |
| Total association ^a^ | Ref | 1.01[0.86, 1.20] | 1.58[1.12, 2.22] |  | Ref | 1.26[1.03, 1.55] | 1.81[1.42, 2.31] |  | Ref | 0.87[0.76, 1.01] | 1.04[0.80, 1.35] |
| Natural direct association |  | 1.01[0.85, 1.20] | 1.57[1.09, 2.25] |  |  | 1.22[0.98, 1.52] | 1.81[1.40, 2.34] |  |  | 0.87[0.76, 1.01] | 1.03[0.79, 1.35] |
| Natural indirect association |  | 1.01[1.00, 1.01] | 1.01[0.97, 1.05] |  |  | 1.03[1.01, 1.05] | 1.00[0.98, 1.03] |  |  | 1.00[1.00, 1.00] | 1.01[1.00, 1.02] |
| Proportion mediated (%) ^b^ |  | NA | 2.6 |  |  | 14.6 | 0.0 |  |  | NA | NA |
| **Reduced HDL** |  |  |  |  |  |  |  |  |  |  |  |
| Total association ^a^ | Ref | 1.03[0.9, 1.19] | 1.51[1.13, 2.02] |  | Ref | 1.50[1.26, 1.78] | 1.62[1.31, 2.00] |  | Ref | 0.86[0.76, 0.97] | 1.06[0.85, 1.32] |
| Natural direct association |  | 1.03[0.89, 1.19] | 1.49[1.10, 2.02] |  |  | 1.49[1.25, 1.77] | 1.60[1.28, 1.99] |  |  | 0.86[0.76, 0.97] | 1.06[0.85, 1.32] |
| Natural indirect association |  | 1.00[1.00, 1.01] | 1.01[0.98, 1.04] |  |  | 1.00[0.99, 1.01] | 1.01[0.99, 1.03] |  |  | 1.00[1.00, 1.00] | 1.00[1.00, 1.00] |
| Proportion mediated (%) ^b^ |  | NA | 3.8 |  |  | 1.4 | 3.2 |  |  | 0.1 | NA |
| **Elevated blood pressure** |  |  |  |  |  |  |  |  |  |  |  |
| Total association ^a^ | Ref | 1.03[0.88, 1.19] | 1.64[1.20, 2.24] |  | Ref | 1.46[1.22, 1.75] | 1.67[1.35, 2.06] |  | Ref | 0.89[0.78, 1.01] | 1.09[0.86, 1.37] |
| Natural direct association |  | 1.02[0.87, 1.19] | 1.64[1.20, 2.24] |  |  | 1.43[1.19, 1.72] | 1.62[1.30, 2.03] |  |  | 0.88[0.77, 1.01] | 1.08[0.86, 1.37] |
| Natural indirect association |  | 1.00[1.00, 1.01] | 1.00[0.99, 1.01] |  |  | 1.02[1.00, 1.04] | 1.03[1.00, 1.06] |  |  | 1.01[1.00, 1.01] | 1.01[1.00, 1.01] |
| Proportion mediated (%) ^b^ |  | NA | 0.4 |  |  | 6.3 | 6.7 |  |  | NA | NA |
| **Hyperglycemia** |  |  |  |  |  |  |  |  |  |  |  |
| Total association ^a^ | Ref | 1.02[0.89, 1.18] | 1.34[0.99, 1.80] |  | Ref | 1.43[1.21, 1.69] | 1.56[1.28, 1.92] |  | Ref | 0.89[0.79, 1.00] | 0.99[0.79, 1.23] |
| Natural direct association |  | 1.02[0.89, 1.17] | 1.32[0.98, 1.79] |  |  | 1.38[1.16, 1.65] | 1.47[1.18, 1.83] |  |  | 0.89[0.79, 1.00] | 0.97[0.78, 1.21] |
| Natural indirect association |  | 1.00[1.00, 1.01] | 1.01[1.00, 1.02] |  |  | 1.03[1.01, 1.05] | 1.06[1.02, 1.11] |  |  | 1.00[1.00, 1.00] | 1.02[1.01, 1.03] |
| Proportion mediated (%) ^b^ |  | NA | 4.0 |  |  | 10.7 | 16.8 |  |  | 1.1 | NA |
| **Hyperuricemia** |  |  |  |  |  |  |  |  |  |  |  |
| Total association ^a^ | Ref | 1.02[0.88, 1.17] | 1.47[1.10, 1.97] |  | Ref | 1.49[1.26, 1.77] | 1.59[1.29, 1.95] |  | Ref | 0.87[0.77, 0.99] | 1.07[0.86, 1.33] |
| Natural direct association |  | 1.01[0.88, 1.17] | 1.46[1.08, 1.96] |  |  | 1.48[1.25, 1.76] | 1.55[1.26, 1.92] |  |  | 0.87[0.77, 0.99] | 1.07[0.86, 1.33] |
| Natural indirect association |  | 1.00[1.00, 1.01] | 1.01[1.00, 1.03] |  |  | 1.01[1.00, 1.01] | 1.02[1.00, 1.04] |  |  | 1.00[1.00, 1.00] | 1.00[1.00, 1.00] |
| Proportion mediated (%) ^b^ |  | NA | 3.8 |  |  | 1.8 | 5.6 |  |  | 1.2 | NA |

^a^ The HRs and 95% CI of the total effect, natural direct effect, and natural indirect effect were calculated using the VanderWeele counterfactual-framework approach. ^27,28^ The models were adjusted for sex, age,

race, education levels, household income, socioeconomic status, smoking status, alcohol consumption, physical activity, fruit and vegetable intake, red and processed meat intake, sleep time (<8 hours, 8-9hours, ＞9 hours, medications use (aspirin, non-aspirin NSAIDs, and proton pump inhibitors use), total sugar intake, total energy, and mutually adjusted for another two beverages. Total effect estimates may vary between models for different mediators due to differences in the number of missing values for each mediator and exposure-mediator interactions.

^b^ Proportion mediated not given in cases where the null effect (i.e. 1) is contained in the 95% CI of the HR of the total effect.

| Table S8. Sensitivity analyses for associations between consumption of three types of beverages and risk of chronic kidney diseases | | | | |  |
| --- | --- | --- | --- | --- | --- |
|  | Lagging the exposure for 2 years to allow a time window for CKD incidence | Excluding the participants with cardiovascular disease at baseline | Further adjusted for energy from beverages | Further adjusted for eGFR | Competing Risk Analysis using Fine-Gray subdistribution hazard model |
| **Sugar-sweetened beverages** |  |  |  |  |  |
| 0 unit per day | Ref | Ref | Ref | Ref | Ref |
| 0–1 unit per day | 1.04[0.97, 1.11] | 1.04[0.97, 1.12] | 1.03[0.96, 1.11] | 1.04[0.97, 1.12] | 1.02[0.95, 1.09] |
| >1 units per day | 1.46[1.31, 1.63] | 1.41[1.25, 1.59] | 1.35[1.2, 1.52] | 1.41[1.25, 1.59] | 1.36[1.22, 1.51] |
| **Artificially sweetened beverages** |  |  |  |  |  |
| 0 unit per day | Ref | Ref | Ref | Ref | Ref |
| 0–1 unit per day | 1.14[1.06, 1.24] | 1.13[1.03, 1.23] | 1.13[1.04, 1.24] | 1.13[1.03, 1.23] | 1.10[1.02, 1.19] |
| >1 units per day | 1.50[1.34, 1.68] | 1.56[1.38, 1.76] | 1.57[1.39, 1.77] | 1.56[1.38, 1.77] | 1.42[1.27, 1.59] |
| **Natural juices** |  |  |  |  |  |
| 0 unit per day | Ref | Ref | Ref | Ref | Ref |
| 0–1 unit per day | 0.85[0.80, 0.90] | 0.85[0.80, 0.91] | 0.84[0.79, 0.90] | 0.85[0.80, 0.91] | 0.88[0.83, 0.93] |
| >1 units per day | 1.00[0.89, 1.12] | 0.99[0.87, 1.12] | 0.95[0.83, 1.07] | 0.99[0.87, 1.12] | 1.01[0.90, 1.13] |

Estimated effects were based on the fully adjusted model (see the footnote in Table 2).

| Table S9. Associations between consumption of three types of beverages and risk of acute kidney injury | | | | |
| --- | --- | --- | --- | --- |
|  | Cases/ Person-years | Hazard Ratio [95% Confidence Interval] | | |
|  |  | Age and gender-stratified model | Multivariable adjusted model 1^†^ | Multivariable adjusted model 2^‡^ |
| **Sugar-sweetened beverages** |  |  |  |  |
| 0 unit per day | 2868/1367675.9 | Ref | Ref | Ref |
| 0–1 unit per day | 1232/526717.1 | 1.18[1.11, 1.27] | 1.15[1.08, 1.23] | 1.17[1.09, 1.25] |
| >1 units per day | 326/136784.1 | 1.53[1.37, 1.72] | 1.31[1.17, 1.47] | 1.36[1.20, 1.53] |
| P for trend |  | <0.001 | <0.001 | <0.001 |
| **Artificially sweetened beverages** | |  |  |  |
| 0 unit per day | 3408/1609314.8 | Ref | Ref | Ref |
| 0–1 unit per day | 705/310513 | 1.31[1.21, 1.42] | 1.21[1.11, 1.31] | 1.18[1.09, 1.28] |
| >1 units per day | 313/111349.4 | 1.95[1.74, 2.19] | 1.58[1.41, 1.78] | 1.55[1.38, 1.74] |
| P for trend |  | <0.001 | <0.001 | 0.0044 |
| **Natural juices** |  |  |  |  |
| 0 unit per day | 2303/982272.2 | Ref | Ref | Ref |
| 0–1 unit per day | 1826/900895.6 | 0.79[0.74, 0.84] | 0.91[0.86, 0.97] | 0.92[0.87, 0.98] |
| >1 units per day | 297/148009.4 | 0.84[0.75, 0.95] | 0.97[0.86, 1.09] | 1.02[0.9, 1.15] |
| P for trend |  | <0.001 | 0.036 | 0.152 |

† Multivariable adjusted model 1: additionally adjusted for race, education levels, household income, socioeconomic status, smoking status, alcohol consumption, physical activity, fruit and vegetable intake, red and processed meat intake, sleep time (<8 hours, 8-9hours, ＞9 hours, medications use (aspirin, non-aspirin NSAIDs, and proton pump inhibitors use) and medications use (aspirin, non-aspirin NSAIDs, and proton pump inhibitors use).
‡ Multivariable adjusted model 2: additionally adjusted for total sugar intake, total energy, and mutually adjusted for another two beverages.


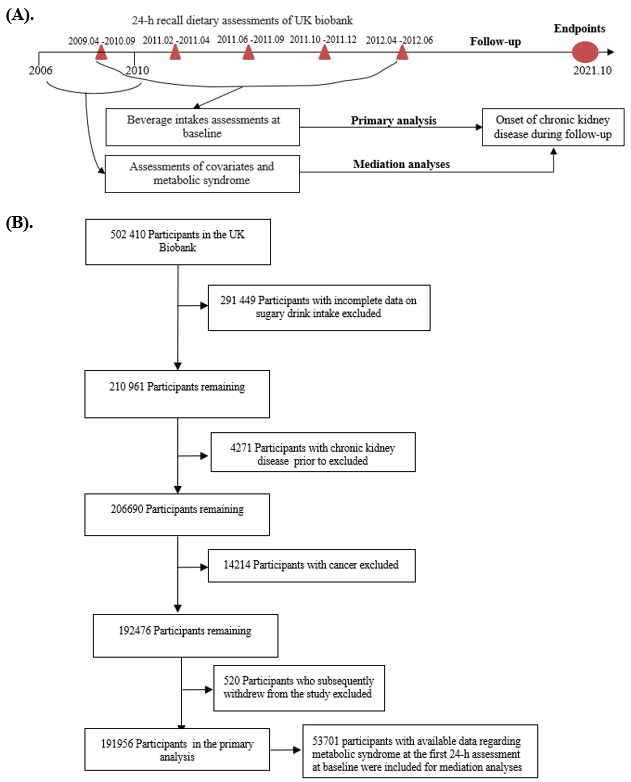


**Figure S1** Diagram of study design and selection process.

1. The concept framework of the study design. (b) The flow chart of the selection process


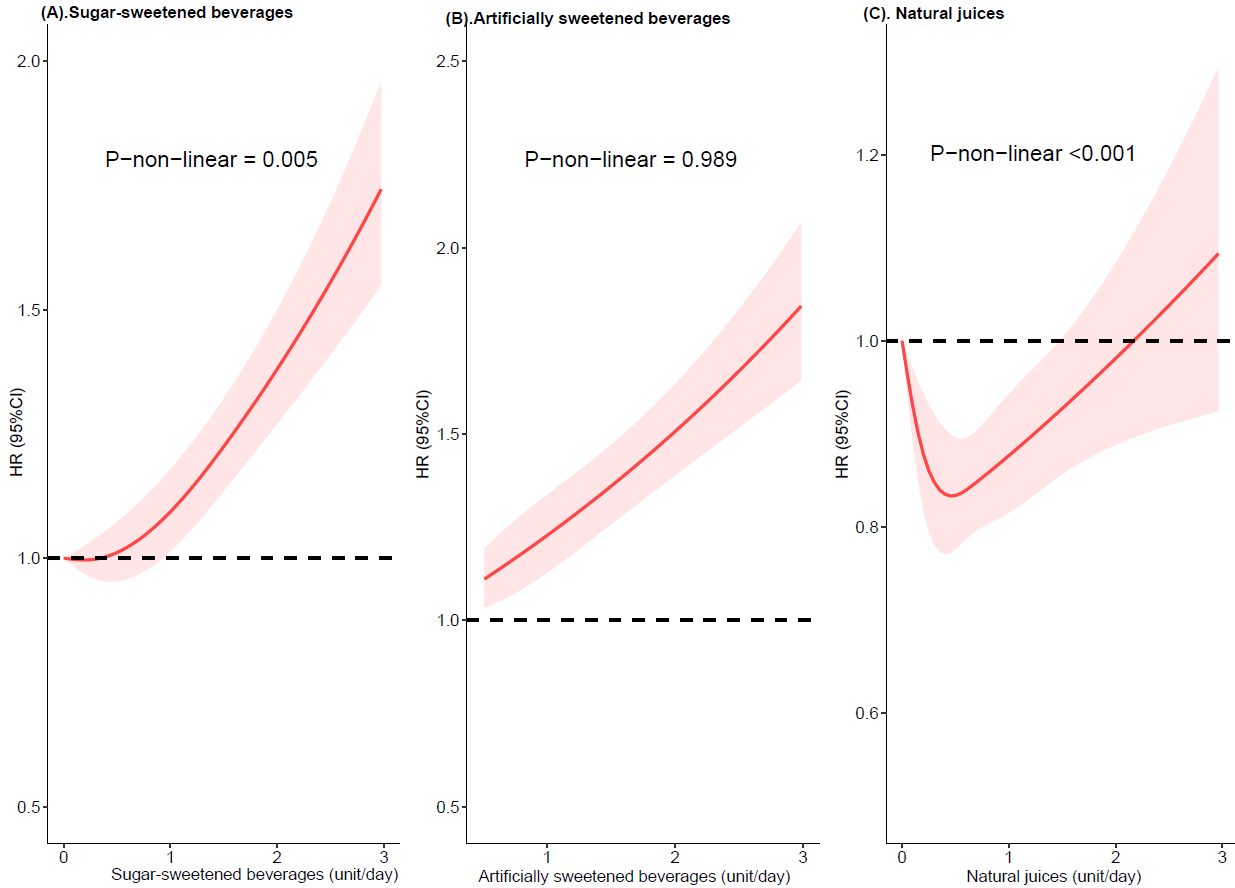


**Figure S2: Risk of incident chronic kidney diseases according to consumption of sugar-sweetened beverages, artificially sweetened beverages and natural juices**

Restricted cubic splines were plotted for participants consuming less than 3 units/day of sugary beverages. HRs were calculated from Cox proportional hazard models adjusted for sex, age, race, education levels, household income, socioeconomic status, smoking status, alcohol consumption, physical activity, fruit and vegetable intake, red and processed meat intake, sleep time, medications use (aspirin, non-aspirin NSAIDs, and proton pump inhibitors use), total sugar intake, total energy, and mutually adjusted for another two beverages.
